# Right Ventricular Structure and Function in Young Adults Born Preterm at Very Low Birth Weight

**DOI:** 10.3390/jcm10214864

**Published:** 2021-10-22

**Authors:** Charlotte Greer, Sarah L. Harris, Richard Troughton, Philip D. Adamson, John Horwood, Chris Frampton, Brian A. Darlow

**Affiliations:** 1 Department of Cardiology, Christchurch Hospital, Christchurch 8011, New Zealand; richard.troughton@cdhb.health.nz (R.T.); philip.adamson@cdhb.health.nz (P.D.A.); 2 Department of Paediatrics, University of Otago, Christchurch 8011, New Zealand; sarah.harris@otago.ac.nz (S.L.H.); brian.darlow@otago.ac.nz (B.A.D.); 3 Department of Medicine, University of Otago, Christchurch 8011, New Zealand; chris.frampton@otago.ac.nz; 4 British Heart Foundation Centre for Cardiovascular Science, University of Edinburgh, Edinburgh EH16 4TJ, UK; 5 Department of Psychological Medicine, University of Otago, Christchurch 8011, New Zealand; john.horwood@otago.ac.nz

**Keywords:** cardiovascular risk, low birth weight, preterm birth

## Abstract

Being born preterm (PT, <37 weeks gestation) or at very low birth weight (VLBW, <1500 g) is associated with increased rates of cardiopulmonary disorders in childhood. As survivors age, late cardiac effects, including right ventricular (RV) remodelling and occult pulmonary hypertension are emerging. In this population-based study, we aimed to investigate right heart structure and function in young adults born PT at VLBW compared to normal-weight term-born controls. The New Zealand VLBW Study has followed all infants born in 1986 with birth weight <1500 g. All were born preterm from 24 to 37 weeks. A total of 229 (71% of survivors) had echocardiograms aged 26–30 years which were compared to age-matched, term-born, normal-weight controls (*n* = 100). Young adults born preterm at very low birth weight exhibited smaller RV dimensions compared to term-born peers. Standard echocardiographic measures of RV function did not differ, but mildly reduced function was detected by RV longitudinal strain. This difference was related to birth weight and gestational age but not lung function or left ventricular function. Echocardiographic strain imaging may be an important tool to detect differences in RV function preterm and VLBW.

## 1. Introduction

Advances in neonatal care over the last 30 years have led to significantly increased survival rates for infants born preterm (<37 weeks gestation), especially in those born very preterm (<32 weeks gestation) or very low birth weight (VLBW <1500 g) [1,2]. In countries with developed health care systems, current survival to discharge ranges from 40 to 55% for those born <24 weeks gestation, to over 95% for those born at 28 weeks. Survival for those weighing 500–1500 g is 85% [3,4,5]. The first generation with these high survival rates is now approaching middle age and the long-term impact of premature birth on non-communicable diseases, including cardiovascular disease, is becoming evident [6].

Adults born very prematurely or with VLBW experience increased rates of health and neurodevelopmental problems compared to term-born, normal-weight peers, particularly cardiopulmonary disorders [1,7,8,9]. Evidence is accumulating of a unique cardiac phenotype of prematurity. Animal and human autopsy studies have shown differences in cardiomyocyte proliferation [10] and ventricular hypertrophy [11]. Multimodality imaging studies have demonstrated differences in left heart structure and function for preterm or low birth weight individuals from the neonatal period to young adulthood [12,13,14,15,16,17] and impaired response to physiological stress [18,19]. Although the magnitude of these differences is small there is concern that over time the preterm heart may be more vulnerable to cardiovascular disease. Longitudinal cohort and population registry studies support this concern, revealing increased rates of heart failure [20,21], ischaemic heart disease [22] and hypertension [23].

To date, evaluation of preterm/low birth weight cohorts has mostly focussed on left heart structure and function. The right ventricle (RV) has been less studied but may be particularly vulnerable to maladaptive responses as the immature heart transitions early from the fetal to neonatal circulation and due to the impact of the chronic lung disease of prematurity and pulmonary vascular disease [19].

The population-based New Zealand VLBW Study comprises a unique cohort that included all VLBW infants born in New Zealand in 1986 [24]. All were born preterm. Here, we report the results of right heart evaluation using echocardiography, including strain imaging, at 26–30 years compared with healthy term born controls. We hypothesized that, VLBW adults would show significant differences in right heart structure and right heart function compared to normal-weight, term-born peers. We also aimed to investigate any associations between perinatal factors, pulmonary function in adulthood and right heart structure and function.

## 2. Materials and Methods

### 2.1. Study Population

The New Zealand Very Low Birth Weight Study is a prospective, population-based cohort study that included all 413 VLBW (<1500 g) infants born in New Zealand in 1986 who were admitted to a neonatal unit [25]. The 338 (82%) who survived to discharge were further assessed at age 7–8 years, and at 22–23 years. At age 26–30 years 250 (77%) of survivors participated in a follow up study and 229 (71%) underwent a comprehensive assessment over 2 days between February 2013 and June 2016. To allow comparison, 100 age-matched, term-born, normal-weight controls were recruited via random sampling from the electoral roll or nomination by a cohort member, with efforts made to assure balance with respect to sex and ethnicity. A flow diagram of recruitment and retention has been previously published (Supplementary Figure S1).

### 2.2. Study Visits

Perinatal and demographic information was collected at birth for the preterm cohort and in young adulthood for control. This included incidence of bronchopulmonary dysplasia (BPD)—defined at that time as persistent oxygen requirement at 36 weeks post-menstrual age. For this evaluation, participants underwent comprehensive assessment including health and social functioning history, anthropometry, pulmonary function testing and transthoracic echocardiography.

### 2.3. Transthoracic Echocardiography

Echocardiography was performed using an iE33 machine (Phillips Healthcare, Amsterdam, The Netherlands) by an experienced cardiac ultrasonographer blinded to study group. Image acquisition and measurements were performed in accordance with American Society of Echocardiography guidelines [26]. Indices of left heart structure and function have been previously reported [14]. Echocardiogram analysis was performed by an assessor blinded to study grouping. Measurements of right heart structure and function were undertaken for this analysis including RV basal, mid and longitudinal dimensions; RV end-diastolic and end-systolic areas; RV wall thickness; right atrial (RA) area and volume; fractional area change (FAC); tricuspid annular plane systolic excursion (TAPSE). Tissue Doppler indices of: tricuspid annular peak systolic velocity (TAPSV); right ventricular index of myocardial performance (RIMP); RV tricuspid lateral annular diastolic velocities of early filling (e’) and atrial contraction (a’) and their ratio; inferior vena cava (IVC) diameter. Volume and area measurements were indexed to body surface area (BSA) determined according to the Mostellar equation, in line with American society of Echocardiography guidelines [27]. Where image quality was judged by the assessor to be insufficient for analysis no measurement was recorded.

### 2.4. Assessment of Right Ventricular Strain

Right ventricular systolic function by myocardial deformation was assessed using TomTec 2D Cardiac Performance Analysis (TomTec Imaging systems, Munich, Germany). If images were of insufficient technical quality for strain assessment (for example, incomplete inclusion of the whole RV), strain was not analysed. For adequate images, end systolic endocardial and epicardial borders of the right ventricle were traced manually. After software speckle tracking, automated end-diastolic borders were reviewed and adjusted if appropriate. Endocardial strain measurements were recorded for the freewall alone and for the whole RV including interventricular septum (global longitudinal strain). Images were analysed by a single assessor blinded to study group. For intraobserver variability, intra-class correlation coefficient was 0.95 indicating excellent reliability.

### 2.5. Lung Function Testing

Lung function data have been reported previously and showed that the PT/VLBW young adults had a higher incidence of airflow obstruction with significantly lower mean forced expiratory volume in 1 s (FEV1), FEV1/FVC (forced vital capacity) ratio and decreased diffusing capacity of the lung for carbon monoxide (DLCO) [28]. In this study we selected FEV1, FEV1/FVC and DLCO to explore associations between right heart structure and lung function.

### 2.6. Statistical Analysis

Dichotomous variables are summarised as frequencies and percentages. The distributions of continuous variables were assessed by visual assessment of density and quantile-quantile plots and are summarised as the mean +/− standard deviation for normally distributed data. Differences in demographic and perinatal data between controls and VLBW groups were explored using independent Student’s *t*-tests and Chi-square tests as appropriate. Sex differences in echocardiographic indices are well described, therefore comparisons were adjusted for sex with analysis of variance (ANOVA). The differences between controls and VLBW, gestational age groups and birth weight groups, and the RV indices or strain, were assessed using ANCOVA with sex included in the models. To further assess the impact of birthweight and gestational age on RV indices and strain, subjects were grouped according to World Health Organization defined sub-categories of prematurity: extremely premature (<28 weeks) very premature (28–31 weeks) and moderate to late preterm (32–37 weeks), and birthweight: VLBW (<1500 g) and extremely low birthweight (<1000 g) [29]. Independent *t*-tests were used to explore the effect of perinatal factors, antenatal steroid use and a diagnosis of BPD, within the study group. The relationships between strain and adult lung function and previously reported left ventricular (LV) indices of ejection fraction (EF), LV volumes and LV mass index were explored using Pearson’s correlation coefficients. Statistical analysis was carried out with IBM SPSS version 25.0, Armonk, NY, USA. A two-tailed *p*-value < 0.05 was taken to indicate statistical significance.

## 3. Results

### 3.1. Participant Characteristics

Table 1 shows demographic and perinatal data. Amongst the VLBW group, 28% had a birth weight <1000 g and 20% had BPD. Over half had exposure to antenatal steroids (ANS). There were no differences between the study and control group for age, ethnicity and sex. PT/VLBW participants were smaller than their peers as young adults.

### 3.2. Echocardiographic Outcomes

#### 3.2.1. Structure

Young adults born at VLBW had smaller right atrial area and volume, smaller RV end-diastolic area and smaller ventricular dimensions (Table 2). Right ventricular wall thickness, as an indicator of hypertrophy, did not differ between groups.

#### 3.2.2. Function

For the functional measures of RV FAC, 2D TAPSE and RIMP there were no differences (Table 2). Strain analysis revealed lower longitudinal RV freewall strain in VLBW participants compared to controls with an absolute difference of approximately 1% between groups. Myocardial deformation, as measured by strain imaging was analysable in 55% of participants. Demographics were similar in those who had strain measured to those who did not. Perinatal characteristics differed slightly: Those who had strain measured had higher birth weight (1.18 kg vs. 1.09 kg) and higher mean gestational age (29.7 vs. 28.8 weeks) at birth. There was no difference in lung function or LVEF or mass but the group with measurable strain did show slightly increased left ventricular volumes (End-diastolic volume index 61.2 vs. 58 cm^3^/m^2^) (Supplementary Table S1).

### 3.3. Impact of Perinatal Risk Factors

#### 3.3.1. Gestational Age

**Figure 1:**
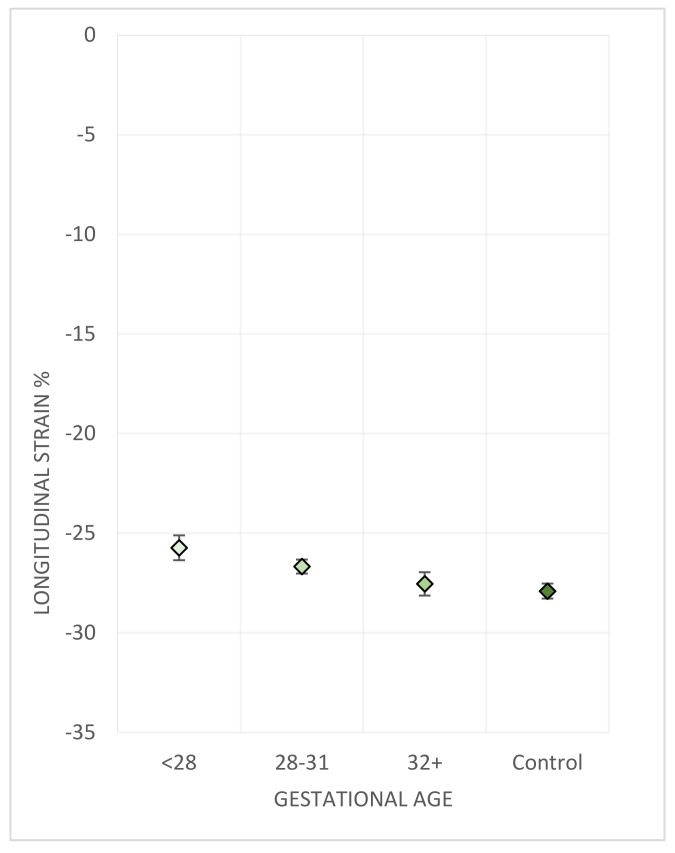
RV freewall strain is associated with gestational age. Right ventricular freewall longitudinal strain compared between gestational age groups corresponding to extremely preterm (<28 weeks), very preterm (28–31 weeks), and moderately to late preterm (32–36 weeks), controls are all term-born [29]. Diamonds indicate the sex-adjusted mean. Bars indicate the standard error.

A gradient of difference in RV dimensions and in longitudinal strain was observed when individuals were grouped according to WHO categories of gestational age (Table 3) (Figure 1).

#### 3.3.2. Birth Weight

**Figure 2:**
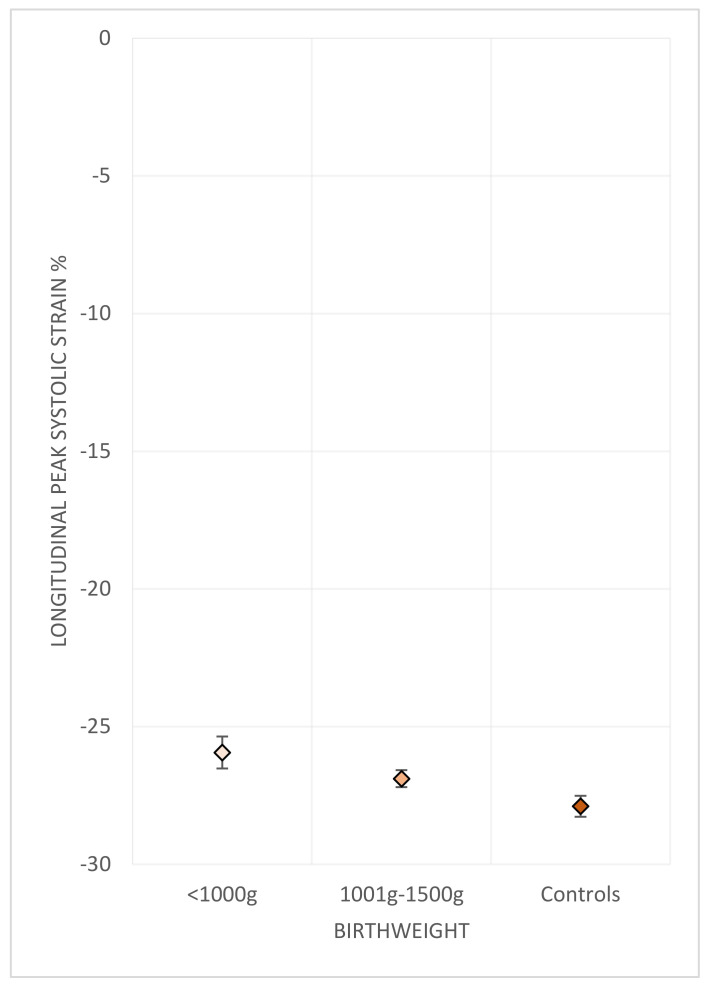
RV freewall strain associated with birth weight. RV longitudinal freewall strain compared between categories of extremely low birth weight (ELBW, <1000 g) and VLBW (<1500 g). Diamonds indicate the sex-adjusted mean. Bars indicate the standard error.

A similar gradient of difference was also observed across groups categorised by birth weight (Table 4) (Figure 2).

#### 3.3.3. Bronchopulmonary Dysplasia

FAC was smaller in VLBW with BPD (40% vs. 43% in no BPD, *p* = 0.025). No difference was found in any other parameter between VLBW with BPD compared to those without BPD (Supplementary Table S2).

#### 3.3.4. Antenatal Steroid Exposure

No difference in right heart indices was found between those exposed and those not exposed to antenatal steroids (Supplementary Table S3).

### 3.4. Lung Function in Adulthood

Data regarding lung function have been previously published [28]. There was no correlation between RV freewall longitudinal strain and lung function as evaluated by FEV1 (r = −0.002, *p* = 0.976); FEV/FVC (r = 0.095, *p* = 0.214); or DLCO (r = −0.034, *p* = 0.661) z-scores.

### 3.5. Relationship to Left Ventricular Indices

No significant association was found between RV strain and left ventricular ejection fraction (r = 0.15, *p* = 0.045), LV end-diastolic volume (r = −0.03, *p* = 0.658), LV end-systolic volume (r = −0.1, *p* = 0.23) or LV mass index (r = −0.02, *p* = 0.75)

## 4. Discussion

In this study, involving a unique population-based cohort of young adults born preterm at VLBW, we have identified measurable differences in right heart structure and function as compared to normal-weight, term-born controls. We confirm the decreased right ventricular end-diastolic chamber size previously reported in existing literature. In addition, we report for the first time, relatively decreased myocardial deformation in VLBW/preterm young adults compared to their peers as determined from evaluation of echocardiographic strain. This was associated with gestational age and birth weight, but not with BPD in infancy or adult lung function.

Smaller end-diastolic RV chamber size in this analysis is consistent with our previously published left heart data from this cohort, which showed smaller LV volumes when indexed to BSA [14]. Using RV wall thickness as an estimate of hypertrophy we found no difference in VLBW adults. There have been conflicting results reported regarding ventricular mass in VLBW and preterm adults [13,14,16]. Animal and autopsy studies and early imaging of preterm infants have demonstrated increased cardiomyocyte hypertrophy but decreased cardiomyocyte endowment [10,11,12,30]. Premature exposure to the extra-uterine environment halts the cardiomyocyte proliferation of the third trimester [31] with limited capacity for cardiomyocyte regeneration after this period. This early switch to the postnatal hypertrophic pattern of cardiomyocyte development and early exposure to the changed haemodynamic load could feasibly result in higher or lower RV mass depending on the balance of circumstances.

Although right ventricular systolic strain has previously been reported using CMR, we report for the first time decreased RV strain measured by echocardiography in a population-based cohort of preterm or VLBW young adults. Our finding of subtly altered strain in VLBW vs. controls is in agreement with the CMR findings of Lewandowski et al. albeit to a lower degree of magnitude. Further analysis of strain and structural measures revealed that differences were greater in those born earlier or at lower birth weight, consistent with data published from other cohorts regarding both RV and LV function [17,32].

We did not demonstrate differences in RV function when assessed using conventional methods with the exception of lower FAC observed in those VLBW participants with a history of BPD. Echocardiographic assessment of RV function can be challenging due to the complex morphology and conventional measures are limited by image and angle dependence [26]. Strain imaging is angle independent and provides superior information in a range of conditions [33,34,35]. Although modest, our finding of reduced strain, in the absence of a measurable decrease in function using standard 2D echocardiographic parameters, highlights the importance of echocardiographic strain as an inexpensive, readily available form of assessment and monitoring in VLBW and preterm populations.

Due to improved survival, the long-term consequences of preterm birth are gaining increased recognition with emerging evidence of cardiovascular effects [18,20,22,36]. Importantly, RV dysfunction is independently associated with worse outcomes in adults with a range of cardiopulmonary conditions [37,38] but data regarding the effects of premature birth on the right heart are scarce. Lewandowski et al. studied 102 young adult survivors of a neonatal feeding trial in infants <1850 g and 132 term-born controls using cardiac magnetic resonance (CMR) [39]. They demonstrated right heart remodelling in preterm-born individuals with higher mass, lower volumes and reduced ejection fraction including 6% with mild systolic dysfunction. CMR-assessed strain was reduced in the preterm group. When compared to left heart function in the same group, between group differences were greater for the right heart [13,39]. A multimodality study by Mohamed et al., reported lower RV fractional area change, TAPSE and ejection fraction, and higher RV mass in preterm young adults but did not report strain [40]. Goss et al. studied a small subset of VLBW individuals as adolescents and adults using CMR [15]. Similar to Lewandowski et al., they found smaller ventricular volumes but, in contrast, no difference in mass or ejection fraction, and found higher longitudinal strain in VLBW adults but not adolescents.

All infants undergo extensive remodelling of the right ventricle after birth as the patent ductus arteriosus closes and the right ventricle changes from a high to low pressure circulation. Being born prematurely disrupts normal cardiac development and this remodelling must occur earlier. In addition cardiac remodelling in preterm infants may also be affected by accompanying abnormal lung development, characterised by a paucity of pulmonary vasculature, dysmorphic and dysfunctional vessels leading to occult pulmonary vascular disease in adulthood [41]. Goss et al. compared 11 VLBW asymptomatic preterm-born young adults with controls and report elevated resting pulmonary pressures via right heart catheterisation, increased pulmonary vascular resistance (PVR) and early RV dysfunction with impaired response to exercise stress [19]. Mohamed et al. [40] measured pulmonary artery acceleration time (PAAT) as an indicator of PVR and found this was lower in preterm young adults but remain coupled to the RV with concordantly lower TAPSE. We were unable to measure PVR in our cohort, but 20% of the NZ VLBW study had chronic lung disease in infancy, associated with a higher incidence of airflow obstruction, gas trapping, and reduced gas exchange young adults [28]. In our cohort, adult lung function and diagnosis of BPD were not found to correlate significantly with strain. This may indicate heterogeneity in the origin of the difference in RV strain in VLBW/PT individuals, with intrinsic myocardial differences as well as afterload related compensatory changes potentially contributing to altered RV structure and function. Right heart function is also commonly impacted by left heart function. Left heart structural and function data have previously been published for this cohort, demonstrating smaller volumes but no difference in function [14]. There was also no relationship between RV strain and LV volumes or ejection fraction and so the subtle difference found in RV strain do not appear to be due to ventricular-ventricular interactions.

Our study has limitations. Not all images were suitable for strain analysis—due mainly to the post hoc nature of this analysis and the LV focussed echo protocol that inadequately captured the RV. Suboptimal acoustic window also limited some strain analysis. Although participants with analysable strain images exhibited demographic characteristics similar to the PT/VLBW cohort as a whole, it is possible, that reduced acoustic windows may have excluded some subjects with RV dysfunction related to pulmonary disease. The strain-analysable group also had slightly higher birth weight and gestational age, and larger LV volumes. However, cardiac dysfunction associated with preterm birth has thus far been associated with *lower* birth weight and gestational age, and *smaller* chamber volumes. We therefore believe that these differences in the strain-analysable group are more likely to have resulted in the under rather than overestimation of reduction in strain in our VLBW cohort, if at all. Our cohort was recruited based on being born VLBW rather than gestational age as was accepted practice at the time. This means the cohort likely represents all the very preterm infants born in New Zealand in 1986. There was unsurprisingly, an association between gestational age and birth weight within the very low birth weight cohort with approximately 2/3rds of the <1 kg group born at less than 28 weeks gestation but the cohort also includes some moderately and late preterm infants with growth restriction and so findings related to gestational age can only be interpreted in this context.

Strengths of our study include comprehensive cardiovascular assessment in a population-based cohort of PT/VLBW individuals, with a high retention rate. Although secular changes in clinical practice may impact the generalisability of our findings, the cohort were cared for with modern strategies, including antenatal steroids and total parenteral nutrition, and as a population-based cohort are representative of the generation of young adults who are now in their fourth decade and at increased risk of cardiovascular disease. It will be valuable to see if our findings are replicated in population-based cohorts from other countries and settings. Although it is somewhat reassuring that strain values remain in the normal range in PT/VLBW individuals in our study, existing data on cardiovascular function in preterm-born young adults provide strong evidence that there are cellular, structural and functional differences in such individuals leading to a ‘cardiac phenotype of prematurity’ [36] including measurably decreased cardiac reserve. We believe that detectable differences in cardiac structure and function in this population of young relatively healthy individuals, warrants further study. Time will tell how this early alteration in function may progress, or if these individuals are more vulnerable to adverse outcomes of acute or chronic cardiac conditions they may encounter as they age. Globally, an estimated 10% of births are preterm [42]. As the vast majority of these infants now survive to adulthood, understanding the trajectory of cardiac function after preterm birth is crucial to ensure premature birth does not lead to premature death.

## 5. Conclusions

Young adults born preterm at very low birth weight have smaller right ventricular dimensions and subtle differences in function measured by echocardiographic strain. Although the changes in function are small by young adulthood, this growing population warrant further research as they age to determine the trajectory of their cardiac function and understand the mechanisms of these differences and evaluate impact on clinical outcomes.

## Figures and Tables

**Table 1:** Demographics and Perinatal Data.

	PT/VLBW *n* = 229	Controls *n* = 100	*p*
Demographics			
Female *n* (%)	127 (55.5)	63 (63)	0.249
Ethnicity *n* (%)			
Māori/Pacific Island	72 (31.5)	24 (24)	0.24
Asian	3 (1.3)	2 (2)	
European	154 (67.2)	74 (74)	
Age at assessment (years)	28.4 ± 1.1	28.2 ± 0.9	0.114
Height cm	168 ± 9	172 ± 9	<0.001
Weight kg	75 ± 19	81 ± 18	0.005
BMI kg/m^2^	27.4 ± 6.3	26.3 ± 6.2	0.14
BSA m^2^	1.85 ± 0.3	1.96 ± 0.2	<0.001
Smokers *n* (%)	71 (31)	21 (21)	0.06
Antihypertensive treatment *n* (%)	5 (2.2)	0	0.33
Perinatal Characteristics			
Birth weight g	1135 ± 234	3372 ± 565	<0.001
Birth weight <1 kg *n* (%)	63 (27.6)	NA	-
Gestation weeks	29.3 ± 2.5	NA	-
BPD *n* (%)	46 (20)	NA	-
ANS *n* (%)	129 (56.3)	NA	-
Small for gestational age (SGA)	72 (31.4%)	NA	-

Values are the mean +/− standard deviation unless otherwise stated. PT: Being born preterm; VLBW: very low birth weight; BSA: body surface area; BPD: bronchopulmonary dysplasia; ANS: antenatal steroids; BMI: body mass index

**Table 2:** Right Ventricular Structure and Function.

	PT/VLBW *n* = 229	Control *n* = 100	*p*
Structural			
End diastolic area indexed to BSA (cm^2^/m^2^)	11.00 (0.17)	11.7 (0.24)	0.028
End systolic area indexed to BSA (cm^2^/m^2^)	6.37 (0.11)	6.53 (0.16)	0.424
RV basal diameter (cm)	3.07 (0.03)	3.28 (0.05)	<0.001
RV mid-cavity diameter (cm)	3.24 (0.03)	3.36 (0.05)	0.062
RV length (cm)	7.25 (0.05)	7.5 (0.08)	0.009
Right atrial volume indexed to BSA (cm^3^/m^2^)	25.3 (0.46)	28.5 (0.7)	<0.001
Right atrial area indexed to BSA (cm^2^/m^2^)	7.37 (0.1)	7.82 (0.15)	0.01
Right ventricular wall thickness (mm)	3.8 (0.1)	3.8 (0.1)	0.85
Functional			
2D TAPSE (cm)	2.23 (0.02)	2.2 (0.03)	0.36
Tissue doppler TAPSV (cm/sec)	12.6 (0.12)	12.9 (0.18)	0.085
Fractional area change %	42.5 (0.52)	43.8 (0.76)	0.187
RV index of myocardial performance	0.45 (0.01)	0.43 (0.01)	0.27
RV TDI e’/a’	1.38 (0.03)	1.37 (0.05)	0.87
Strain	*n* = 116	*n* = 60	
Longitudinal freewall strain %	−26.7 (0.27)	−27.9 (0.38)	0.01
Global longitudinal strain %	−23.6 (0.27)	−24.9 (0.37)	0.003

Values are the sex-adjusted mean (standard error) unless otherwise stated. RV: right ventricular; RV TDI: right ventricular tissue doppler imaging

**Table 3:** RV structural and strain indices compared by gestational age.

	Gestational Age				*p*
	<28	28–31	32+	Control	
	*n* = 55	*n* = 131	*n* = 41	*n* = 100	
Right Ventricular Structure					
End diastolic area indexed to BSA (cm^2^/m^2^)	10.8 (0.4)	11.0 (0.2)	11.3 (0.4)	11.7 (0.2)	0.13
End systolic area indexed to BSA (cm^2^/m^2^)	6.2 (0.3)	6.4 (0.2)	6.6 (0.3)	6.5 (0.2)	0.55
RV basal diameter (cm)	3.0 (0.06)	3.1 (0.04)	3.2 (0.07)	3.3 (0.05)	0.001
RV mid cavity diameter (cm)	3.2 (0.07)	3.3 (0.05)	3.4 (0.08)	3.4 (0.05)	0.06
RV Length (cm)	7.3 (0.1)	7.2 (0.07)	7.4 (0.12)	7.5 (0.08)	0.05
RA volume indexed to BSA (cm^3^/m^2^)	24.1 (1.0)	25.3 (0.6)	26.9 (1.1)	28.8 (0.7)	<0.001
RA area indexed to BSA (cm^2^/m^2^)	7.2 (0.2)	7.4 (0.12)	7.8 (0.22)	7.8 (0.15)	0.01
Right ventricular wall thickness (cm)	3.9 (0.1)	3.8 (0.1)	3.7 (0.1)	3.8 (0.1)	0.83
Right Ventricular Systolic Strain					
Longitudinal RV freewall strain %	−25.7 (0.6)	−26.7 (0.4)	−27.6 (0.6)	−27.9 (0.4)	0.01
Global longitudinal RV strain %	−22.3 (0.6)	−23.8 (0.3)	−23.9 (0.6)	−24.9 (0.4)	0.004

Values are the sex-adjusted mean (standard error) unless otherwise stated. *p*-values are for birthweight group effect from ANOVA including adjustment for sex.

**Table 4:** RV structural and strain indices compared by birth weight.

	Birth Weight			
	≤1000 g *n* = 63	1001–1500 g *n* = 165	Control *n* = 100	*p*
Right Ventricular Structure				
End diastolic area indexed to BSA (cm^2^/m^2^)	10.4 (0.3)	11.2 (0.2)	11.6 (0.2)	0.012
End systolic area indexed to BSA (cm^2^/m^2^)	6.0 (0.2)	6.5 (0.1)	6.5 (0.2)	0.12
RV basal diameter (cm)	3.0 (0.6)	3.1 (0.4)	3.3 (0.5)	<0.001
RV mid cavity diameter (cm)	3.0 (0.06)	3.3 (0.04)	3.4 (0.05)	<0.001
RV Length (cm)	7.1 (0.1)	7.3 (0.1)	7.5 (0.1)	0.004
Right atrial volume indexed to BSA (cm^3^/m^2^)	23 (0.9)	26.2 (0.5)	28.8 (0.7)	<0.001
Right atrial area indexed to BSA (cm^2^/m^2^)	7.0 (0.2)	7.5 (0.1)	7.8 (0.1)	0.001
Right ventricular wall thickness (mm)	3.8 (0.1)	3.8 (0.1)	3.8 (0.1)	0.988
Right Ventricular Systolic Strain				
Longitudinal freewall strain %	−25.9 (0.6)	−26.9 (0.3)	−27.9 (0.4)	0.012
Global longitudinal strain %	−22.2 (0.6)	−23.9 (0.3)	−24.9 (0.4)	<0.001

Values are the sex-adjusted mean (standard error) unless otherwise stated. *p*-values are for birthweight group effect from ANOVA including adjustment for sex.

## Data Availability

The data presented in this study are available in this article and Supplementary Materials.

## Supplementary Materials

The following are available online at https://www.mdpi.com/article/10.3390/jcm10214864/s1, Figure S1: Flow diagram of recruitment and retention of VLBW participants, Table S1: Demographics, perinatal clinical characteristics and left heart variables in all subjects who had analysable strain compared to those who did not; Table S2: RV structure, function and strain values in PT/VLBW subjects with and without a diagnosis of bronchopulmonary dysplasia; Table S3: RV structure, function and strain values in PT/VLBW subjects with and without exposure to antenatal steroid.
