# Supplementary material for: Right Ventricular Structure and Function in Young Adults Born Preterm at Very Low Birth Weight
Source: J Clin Med. 2021 Oct 22;10(21):4864. doi: 10.3390/jcm10214864 (PMC8584927; doi:10.3390/jcm10214864)
Supplement: Supplementary file 1 [file jcm-10-04864-s001.zip › jcm-1417122-SI.pdf]

Supplemental Data

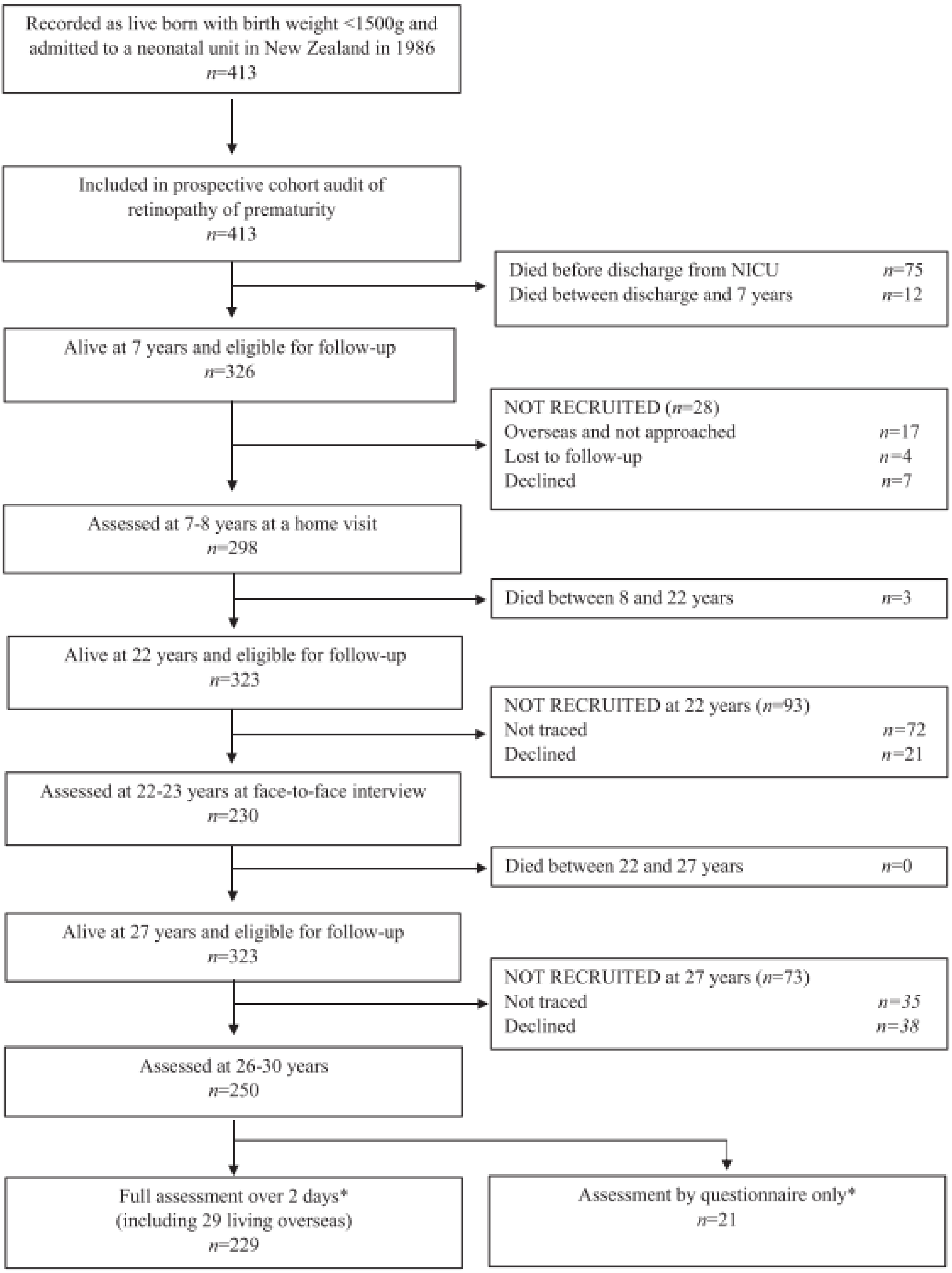

Supplementary figure S1. Flow diagram of recruitment and retention of VLBW participants. (Modified with permission from: Darlow BA, et al. Metabolic syndrome in very low birth weight young adults and controls: the New Zealand 1986 VLBW Study. J Pediatr 2019;206:128-33 133.e1). *Between February 2013 and November 2016. NICU, neonatal intensive care unit.

	Strain<br>n = 176	No strain<br>n = 153	P
**Demographics**			
Age at assessment. years	28.4 ± 1.1	28.2 ± 0.9	0.065*
Ethnicity n (%)<br>Māori/Pacific Island<br>Asian<br>NZ European	 47(26.7)<br>3(1.7)<br>126(71.6)	 49(32)<br>2(1.3)<br>102(66.7)	 0.56
Height cm	169.4±9.0	169.1±9.5	0.88*
Weight, kg	75.3±15.4	78.0±22.3	0.150*
BMI kg/m²	26.2±5.3	27.2±7.2	0.210*
BSA m²	1.9±0.2	1.9±0.3	0.286*
Smokers n (%)	45(27)	46(30)	0.41
**Perinatal Characteristics**			
Birth weight g	1177±226	1091±236	0.05*
Birth weight <1kg n (%)	26(15)	37(24)	0.03
Gestation weeks	29.7±2.6	28.8±2.3	0.005*
BPD n (%)	23(20)	23(21)	0.89
ANS n (%)	50(43)	49(44)	0.92
**Left Heart Function**			
LV Ejection Fraction %	64.1±4.2	64.3±4.5	0.731*
End-diastolic volume, indexed to BSA (cm^3/m^2)	61.2±11.5	57.8±11.3	0.01*
End-systolic volume, indexed to BSA (cm^3/m^2)	22±5.3	20.6±5.1	0.02*
LV mass, indexed to BSA (g/m^2)	92.6±21.2	89.7±19.3	0.28*
**Lung Function**			
FEV1 z score	- 0.46±1.13	-0.55±1.3	0.515*
FEV1/FVC z score	- 0.98±1	-1.1±1.2	0.336*
DLCO z score	-0.53±1	-0.53±1	0.99*

Supplementary Table S1. Demographics, perinatal clinical characteristics and left heart variables in all subjects who had analysable strain compared to those who did not.

Values are unadjusted mean ± standard deviation unless otherwise stated.

ANS -exposure to antenatal steroids; BMI indicates body mass index; BPD - bronchopulmonary dysplasia; BSA – body surface area; DLCO –Diffusing capacity for carbon monoxide; FEV1 – forced expiratory volume in 1 second; FVC – forced vital capacity; LV – left ventricle;

*p value adjusted for sex

	BPD	No BPD	P
n*	46 (20)	182 (80)	
**Right Ventricular Structure**			
End diastolic area index cm²	11.5 (0.4)	10.9 (0.2)	0.2
End systolic area index cm²	6.85 (0.26)	6.27 (0.12)	0.04
RV basal diameter cm	3.1 (0.1)	3.07 (0.03)	0.69
RV mid cavity diameter cm	3.2 (0.08)	2.26 (0.04)	0.51
RV Length cm	7.27 (0.11)	7.25 (0.06)	0.92
Right atrial volume index cm²	24.37 (0.97)	25.54 (0.5)	0.29
Right atrial area index cm²	7.36 (0.21)	7.39 (0.11)	0.92
Right ventricular wall thickness mm	0.36 (0.01)	0.38 (0.01)	0.12
**Right Ventricular Function**			
TAPSE cm	2.17 (0.03)	2.25 (0.03)	0.08
Tissue doppler tricuspid lateral annular systolic velocity cm/sec	12.62 (0.26)	12.54 (0.13)	0.78
Fractional Area Change %	40.07 (1.2)	43.07 (0.56)	0.03
RV myocardial performance index	0.47 (0.02)	0.44 (0.01)	0.15
RV TDI e'/a'	1.46 (0.07)	1.36 (0.04)	0.25
End diastolic LV eccentricity index	1.04 (0.01)	1.03 (0.01)	0.52
End systolic LV eccentricity index	1.022 (0.01)	1.013 (0.004)	0.4
**Right Ventricular Systolic Strain**			
n	23	93	
Longitudinal endocardial freewall strain %	-26.3 (0.3)	-26.76 (0.6)	0.49
Global longitudinal endocardial strain %	-23.04 (0.56)	-23.67 (0.28)	0.32
Global radial strain %	-26.9 (2.81)	-29.8 (1.4)	0.36

Supplementary table S2. RV structure, function and strain values in PT/VLBW subjects with and without a diagnosis of bronchopulmonary dysplasia (BPD)

Values are sex adjusted mean (standard error) unless otherwise stated.

RV indicates right ventricle; LV Left ventricle

RV TDI e'/a' indicates right ventricular tissue doppler early (e')/ atrial (a') filling velocity ratio

*total number VLBW participants but numbers for echocardiographic measurements ranged from 20 - 46 in those with BPD diagnosis and 130 – 182 in those without.

	ANS	No ANS	P
*n (%)	129 (56)	99 (43)	
**Right Ventricular Structure**			
End diastolic area index cm^2	10.9	11.2	0.34
End systolic area index cm^2	6.32	6.44	0.58
RV basal diameter cm	3.04	3.12	0.2
RV mid cavity diameter cm	3.24	3.25	0.9
RV Length cm	7.26	7.26	0.99
Right atrial volume index cm^2	25	25.7	0.41
Right atrial area index cm^2	7.3	7.49	0.34
Right ventricular wall thickness mm	0.38	0.38	0.87
**Right Ventricular Function**			
TAPSE cm	2.2	2.3	0.19
Tissue doppler tricuspid lateral annular systolic velocity cm/sec	12.4	12.8	0.09
Fractional Area Change %	42	43.2	0.25
RV myocardial performance index	0.46	0.43	0.1
RV TDI e'/a'	1.36	1.41	0.45
End diastolic LV eccentricity index	1.04	1.03	0.41
End systolic LV eccentricity index	1.02	1.02	0.28
**Right Ventricular Systolic Strain**			
n	66	50	
Longitudinal endocardial freewall strain %	-26.2	-27.2	0.07
Global longitudinal endocardial strain %	-23.4	-23.8	0.41
Global radial strain %	-30.4	-27.7	0.6

Supplementary table S3. RV structure, function and strain values in PT/VLBW subjects with and without exposure to antenatal steroid (ANS)

Values are sex adjusted mean unless otherwise stated.

RV indicates right ventricle; LV – left ventricle

RV TDI e'/a' indicates right ventricular tissue doppler early (e')/ atrial (a') filling velocity ratio

*Total number VLBW participants but numbers for echocardiographic measurements ranged f81-127 in those who were exposed to ANS and 67-99 in those who weren't.
